# Bridging Gaps in Psychiatric Training Across Borders: Design and Delivery of an International Webinar Series

**DOI:** 10.1192/bjo.2026.11279

**Published:** 2026-06-30

**Authors:** Shehroz Shakeel, Sanaa Moledina

**Affiliations:** 1 Coventry and Warwickshire Partnership Trust, Coventry, United Kingdom; 2 Cambridgeshire and Peterborough NHS Foundation Trust, Cambridge, United Kingdom

## Abstract

**Aims:**

Postgraduate psychiatry training varies globally in clinical competencies, patient exposure, trainee autonomy, and teaching methods. Teaching webinars can address gaps in training needs by facilitating knowledge and skills development, especially in low and middle-income countries. Having completed undergraduate medical training in developing countries and currently practicing psychiatry in the UK, the authors drew upon their direct experience of differing training structures to develop a webinar series. This series was delivered in collaboration with the British Pakistani Psychiatric Association (BPPA) to share key UK training curricula skills and workplace practices with international psychiatry resident doctors with an aim to further develop their clinical practice and improve patient care.

**Methods:**

Seven interactive sessions covered reflective practice and Balint group principles, trauma-informed psychiatry, CBT frameworks, clinical skills (capacity and risk assessments) and ethical considerations of patient confidentiality and consent, designing research, audits, and quality improvement projects, and UK psychiatry career pathways, were delivered by UK-based doctors. Within one week of advertising through BPPA networks, 90 doctors from 19 countries applied (52.2% non-training, 47.8% trainees). Fifty participants were shortlisted based on expressions of interest. Post-series feedback was collected.

**Results:**

The survey response rate was 44%. The series received a mean rating of 4.59/5, with speaker line-up rated 4.55/5, accessibility 4.23/5, and topic relevance 4.68/5. Qualitative feedback reported sessions as highly informative, diverse in topic range, clinically relevant, and presenting concepts that were new to many participants, like Balint groups. Participants recommended more detailed sessions, increased case-based learning, pre-session access to slides and post-session recordings, and more focus on NHS-specific practices versus other healthcare systems. There was strong interest in future sessions with longer duration and increased frequency.

**Conclusion:**

The feedback highlighted significant demand for accessible, high-quality psychiatric education. Topics like reflective practice and trauma-informed care, not universally integrated into existing curricula, generated particular interest. The positive response has motivated the authors to deliver similar initiatives for wider cohorts, incorporating participant feedback to enhance accessibility and educational impact. Such programmes offer structured educational platforms for early-career psychiatrists globally to access skills-focused learning opportunities, while facilitating cross-cultural exchange of psychiatric expertise. While traditional psychiatric education often overlooks cultural nuances in patient care, international collaboration among psychiatrists can improve patient outcomes and reinforce healthcare systems by fostering both cultural competence and cultural humility.

